# 

**Published:** 1873-06

**Authors:** 


### TABLE OF CONTENTS.

### ORIGINAL COMMUNICATIONS.

CLINICAL RESEARCHES IN ELECTRO-THERAPEUTICS. By A. D. ROCKWELL, M.D. . 123

SELECTIONS FROM MY CASE-BOOK. (Illustrating the Efficacy of Iodide Potassium in Asthma, and the Beneficial Effects of Hypodermic Injection of Morphia Directly over the Seat of Pain.) By J. S. TODD, M.D. . . . . . . . . . . 134

REPORT OF CASES. By W. D. HOYT, M.D. . . . . . . . . . . . . . . . . . . . . . . . . . . . . . . . . . . . . 138

A CASE OF DYSMENORRHŒA AND LEUCORRHŒA PRODUCED BY ANTE-FLEXION OF THE UTERUS, ACCOMPANIED BY ENDO-CERVICITIS. By J. T. JELKS, M.D . . . . 145

REPORT OF D. W. HAMMOND, M.D., CHAIRMAN OF THE COMMITTEE ON SURGERY FOR THE EIGHTH CONGRESSIONAL DISTRICT. . . . . . . . . . . . . . . . . . . . . . . . . . . . . . . 147

(Embracing: Cutting Throat—Operation Unsuccessful, and Performed by the Patient. By D. W. Hammond, M.D.; Rapid and Immense Cystic Collection—Ovariotomy—Death. By C. B. Nottingham, M.D.; Tracheotomy. By G. E. Sussdorff, M.D.)

MODIFICATION OF YELLOW FEVER BY PRECEDING DISEASED STATES OF THE SYSTEM. By JOSEPH JONES, M.D . . . . . . . . . . . . . . . . . . . . . . . . . . . . . . . . . . . . . . . 157

TWENTY-FOURTH ANNUAL MEETING OF THE AMERICAN MEDICAL ASSOCIATION . . . 161

### EDITORIAL AND MISCELLANEOUS.

NORMAL OVARIOTOMY . . . . . . . . . . . . . . . . . . . . . . . . . . . . . . . . . . . . . . . . . . . . 175

THE ORIGIN AND NATURE OF CANCER . . . . . . . . . . . . . . . . . . . . . . . . . . . . . . . . . 177

HORSFORD'S ACID PHOSPHATE . . . . . . . . . . . . . . . . . . . . . . . . . . . . . . . . . . . . . . 178

MISTAKING SPONTANEOUS RECOVERIES FOR THERAPEUTICAL RESULTS . . . . . . . . . . . 179

LOCAL USE OF CHLORATE OF POTASSA IN OPEN CARCINOMA BY DR. BUROW, SEN. . 180

NITRIC ACID IN WHOOPING-COUGH . . . . . . . . . . . . . . . . . . . . . . . . . . . . . . . . . . 181

